# Epigenetic regulation and its therapeutic potential in hepatitis B virus covalently closed circular DNA

**DOI:** 10.1016/j.gendis.2024.101215

**Published:** 2024-02-03

**Authors:** Jihua Ren, Shengtao Cheng, Fang Ren, Huiying Gu, Daiqing Wu, Xinyan Yao, Ming Tan, Ailong Huang, Juan Chen

**Affiliations:** a The Key Laboratory of Molecular Biology of Infectious Diseases Designated by the Chinese Ministry of Education, Chongqing Medical University, Chongqing 400000, China; b Chongqing Key Laboratory of Sichuan-Chongqing Co-construction for Diagnosis and Treatment of Infectious Diseases Integrated Traditional Chinese and Western Medicine, Chongqing Hospital of Traditional Chinese Medicine, Chongqing 400000, China

**Keywords:** cccDNA, Epigenetic regulation, HBV, HBx, Transcription

## Abstract

Human hepatitis B virus (HBV) infection is the major cause of acute and chronic hepatitis B, liver cirrhosis, and hepatocellular carcinoma. Although the application of prophylactic vaccination programs has successfully prevented the trend of increasing HBV infection prevalence, the number of HBV-infected people remains very high. Approved therapeutic management efficiently suppresses viral replication; however, HBV infection is rarely completely resolved. The major reason for therapeutic failure is the persistence of covalently closed circular DNA (cccDNA), which forms viral minichromosomes by combining with histone and nonhistone proteins in the nucleus. Increasing evidence indicates that chromatin-modifying enzymes, viral proteins, and noncoding RNAs are essential for modulating the function of cccDNA. Therefore, a deeper understanding of the regulatory mechanism underlying cccDNA transcription will contribute to the development of a cure for chronic hepatitis B. This review summarizes the current knowledge of cccDNA biology, the regulatory mechanisms underlying cccDNA transcription, and novel anti-HBV approaches for eliminating cccDNA transcription.

## Introduction

Although a safe and effective vaccine has been applied to hepatitis B virus (HBV) transmission prevention for more than forty years, chronic HBV infection continues to threaten human health around the world. According to a recent systematic analysis, the global prevalence of HBV infection for people of all ages was 4.1%, which is consistent with the estimate indicating that 316 million people are infected.^1^ In approximately 8%–20% of patients with chronic hepatitis B, the disease progresses to cirrhosis. Among those with cirrhosis, 2%–5% of patients develop hepatocellular carcinoma.^2^ Due to the popularization of HBV vaccination and antiviral treatment, the mortality of HBV-related diseases has decreased. However, the number of HBV-related deaths remains at 555,000 annually.^1^

HBV entry into hepatocytes has been shown to require low-affinity binding of the virus to heparan sulfate proteoglycans and high-affinity binding to the receptor sodium taurocholate cotransporting polypeptide.^3^, ^4^, ^5^ After capsids of the incoming nucleocapsid are uncoated, the relaxed circular DNA (rcDNA) of the virus is transferred to the nucleus and converted into covalently closed circular DNA (cccDNA) minichromosomes, which are assembled with histones and nonhistones.^6^^,^^7^ Then, cccDNA serves as a template for the production of all viral RNA transcripts. In the cytoplasm, the 3.5 kb genomic RNA precursor is encapsidated and retrotranscribed into single-stranded DNA via the action of a viral polymerase, yielding viral genomic rcDNA. The rcDNA-containing nucleocapsid is enveloped in multivesicular bodies and secreted from the hepatocyte as a progeny virion or delivered into the nucleus to increase the pool of cccDNA.^8^^,^^9^ In the HBV life cycle, cccDNA is vital for the amplification of HBV, suggesting that the establishment of the cccDNA pool underlies the major challenge to HBV infection abrogation.

Complete HBV elimination (considered an undetectable level of serum hepatitis B surface antigen (HBsAg) and HBV DNA, cccDNA eradiation, and integrated HBV DNA) is the key to the reduction in the cases of HBV infection-associated liver diseases. However, because a stable reservoir of cccDNA persists in the nucleus, the eradication of HBV is very difficult. Therefore, the current aim of chronic hepatitis B treatment is a functional cure (undetectable HBV DNA and HBsAg with or without HBsAg seroconversion).^10^^,^^11^ To achieve a functional cure, silencing cccDNA transcriptional activity is a promising strategy; however, both currently approved methods, nucleos(t)ide analogues and interferon derivatives (IFNs), do not primarily target HBV cccDNA.^12^ Hence, it is essential to develop a novel strategy for silencing cccDNA transcription. Therefore, this review focuses on the regulation of cccDNA transcription mediated by host factors and viral proteins, as well as recently developed antiviral approaches targeting cccDNA transcription.

## The basic biology of HBV cccDNA

The formation of cccDNA is critical for producing progeny viruses. Although the mechanism permitting the conversion of rcDNA to cccDNA is not clearly understood, recent advances have revealed multistep enzymatic reactions involved in cccDNA generation.^13^ Presumably, tyrosyl-DNA phosphodiesterase 2 removes the viral polymerase^14^; structure-specific endonuclease 1 cleaves the 5′-flap and RNA primer,^15^^,^^16^ activating proliferating cell nuclear antigen, the replication factor C complex, and DNA polymerase, which contribute to the synthesis of the plus strand^16^^,^^17^; and DNA ligase 1/3 and topoisomerases are involved in the ligation of both strands of rcDNA.^18^^,^^19^ Interestingly, the five core components, including proliferating cell nuclear antigen, replication factor C, polymerase δ, structure-specific endonuclease 1, and DNA ligase 1, constitute the smallest set of factors essential and sufficient for DNA lagging strand synthesis during cccDNA formation.^18^ Therefore, cccDNA formation may be a more complex process than is currently realized, and additional host factors may need to be identified.^20^

cccDNA is organized as a nucleosome-decorated minichromosome and is transcribed into viral mRNA via cellular RNA polymerase II.^21^ In 1994, Bock et al first observed the chromatin-like structure, which appeared with the typical “beads-on-a-string” arrangement, and an average of 18 nucleosomes constituted the HBV minichromosome with approximately 180 base pairs of DNA in each nucleosome.^22^ They further identified the nucleosome, with DNA wrapped around histones H2A, H2B, H3, H4, and H1. Moreover, the viral proteins HBc and HBx were found to be components of the HBV minichromosome.^23^^,^^24^ Based on the identified structure, host transcription factors, chromatin-modifying enzymes, chromatin remodellers, and other enzymes bind to cccDNA minichromosomes, enabling cccDNA to yield efficient viral gene expression.^25^, ^26^, ^27^, ^28^, ^29^, ^30^, ^31^, ^32^ In addition, the HBx protein has been confirmed to be essential for activating cccDNA transcription,^23^^,^^33^ but whether HBc is involved in the regulation of cccDNA transcription is controversial.^24^^,^^34^^,^^35^ As a minichromosome existing in the nucleus, cccDNA is inherently stable in the nucleus with a long half-life.^36^^,^^37^ Furthermore, a low copy number (a median of 1.5 copies per cell) of cccDNA in liver biopsy samples from chronic hepatitis B patients has been shown to be sufficient to maintain chronic infection.^38^, ^39^, ^40^, ^41^, ^42^, ^43^ Therefore, understanding the mechanism of the host factors and viral proteins maintaining cccDNA transcriptional activity to developing new strategies for silencing cccDNA is an essential precondition for achieving a functional cure.

## Regulation of cccDNA transcription

### HBV DNA methylation

DNA methylation, a process by which methyl groups are added to cytosine or adenine, plays a crucial role in the regulation of gene expression during the HBV life cycle. As the transcription template of HBV, cccDNA methylation results in HBV transcription repression, decreased viremia, and hepatitis B e antigen (HBeAg) loss.^44^^,^^45^ Performing thorough research, investigators found that cccDNA tends to be methylated at three cytosine-phosphate-guanine I–III (CpG I–III) islands, especially CpG II and III.^46^ Given that CpG II is located upstream of the enhancer II/core gene promoter, methylated CpG II results in decreased pregenomic RNA transcription and thus reduced HBeAg levels.^47^^,^^48^ Moreover, CpG III overlaps with the initial codon of the Sp1 promoter and P gene, and methylation of CpG III is associated with lower HBsAg levels, which is important because HBsAg is critical to long-term infection. Further, methylated CpG III is also closely related to the occurrence and development of liver cancer.^49^ In addition, DNA methyltransferases are involved in cccDNA transcriptional regulation; for example, DNA methyltransferase gene 3A, which can increase the cccDNA methylation rate and decrease HBV RNA levels.^50^ Overall, cccDNA methylation exerts an inhibitory effect on HBV transcription.

### Histone modification of the cccDNA minichromosome

**Figure 1:**
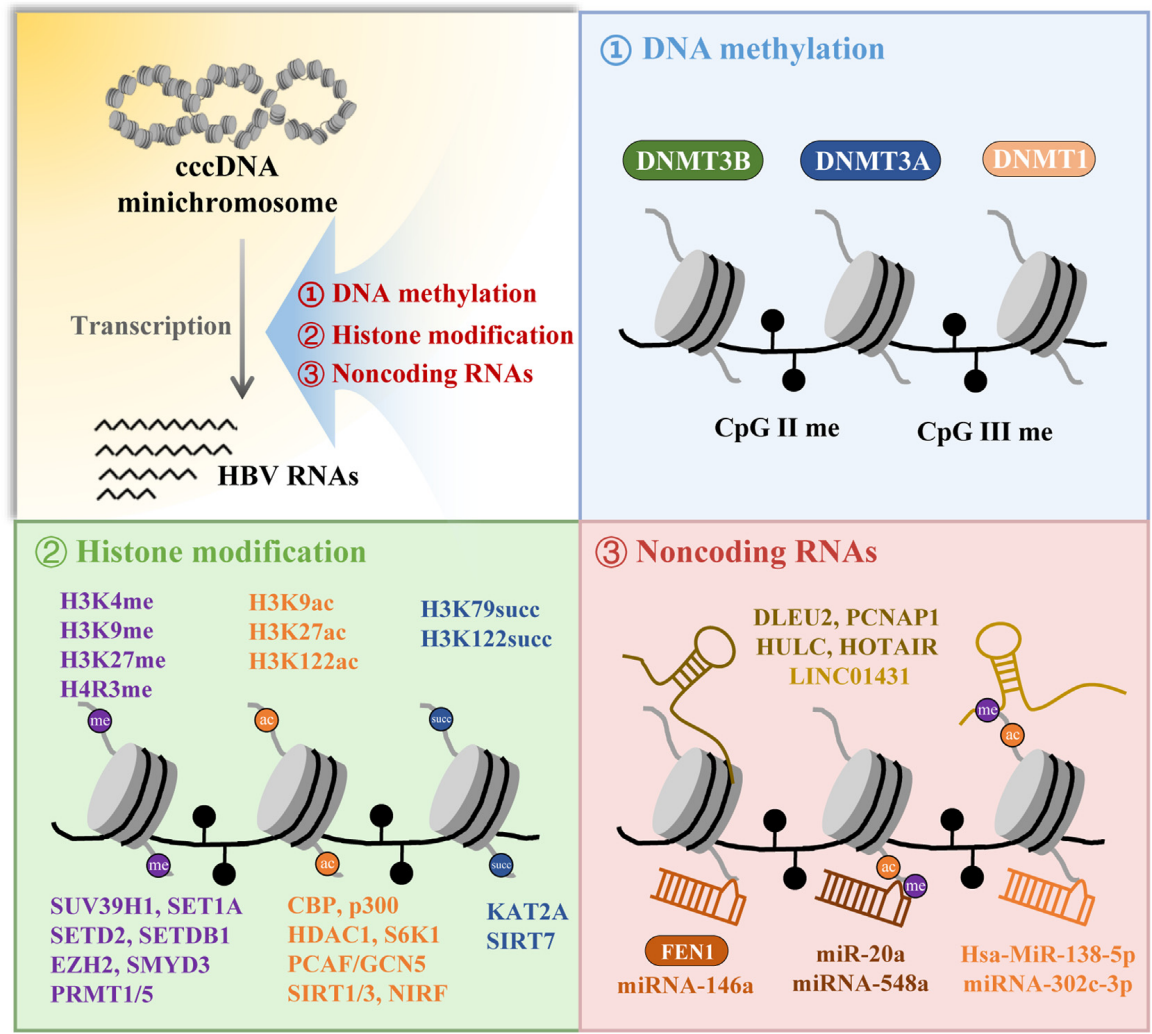
Various mechanisms of cccDNA transcriptional regulation. ① cccDNA transcription is regulated by DNA methyltransferase which could promote cccDNA methylation. ② cccDNA transcription can be controlled by epigenetic modification of histones, such as methylation, acetylation, and succinylation. The correlated methyltransferases, acetyltransferases, and succinyltransferase are listed below, respectively. ③ cccDNA transcription is regulated by non-coding RNAs. The potential non-coding RNAs are provided. cccDNA, covalently closed circular DNA.

Tropberger et al systematically revealed the first genome-wide maps of posttranslational modifications on cccDNA-containing chromatin, with posttranslational modifications found to be distributed nonrandomly across the HBV genome. They found that active transcriptional markers histone H3 lysine 4 trimethylation, histone H3 lysine 27 acetylation, histone 3 lysine 36 trimethylation, and histone H3 lysine 122 acetylation, and repressive transcriptional markers histone 3 lysine 9 trimethylation and histone 3 lysine 27 trimethylation were enriched on cccDNA at different abundance levels.^28^ An increasing number of studies have confirmed that cccDNA transcription can be controlled by epigenetic modifications of histones,^51^ such as methylation, acetylation, and succinylation (Fig. 1). Methylation usually occurs at lysine and arginine residues and is mediated mainly by lysine methyltransferases, lysine demethylases, and arginine methyltransferases. During HBV infection, elevated recruitment of the methyltransferase suppressor of variegation 3–9 homolog 1 and decreased recruitment of SET domain containing 1A histone lysine methyltransferase to cccDNA lead to increased trimethylation of histone H3 lysine K9 and decreased trimethylation of histone H3 lysine K4, which results in cccDNA repression.^52^ In addition, other methyltransferases also participate in cccDNA transcription, such as SET domain-containing 2,^53^ SET domain bifurcated 1,^54^ enhancer of zeste homolog 2,^55^ and SET and MYND domain-containing protein 3.^56^ Notably, dimethylation of arginine 3 on H4 plays a repressive role in cccDNA transcription, which may be mediated by arginine methyltransferase 1^21^ and arginine methyltransferase 5.^26^

In addition to methylation, HBV transcription is strictly regulated by the acetylation status of cccDNA-bound histones. Histone acetylation leads to chromatin decondensation, which makes DNA more accessible to transcription machinery. On cccDNA, high levels of histone H3 lysine 9 acetylation, histone H3 lysine 27 acetylation, and histone H3 lysine 122 acetylation have been found to be closely related to transcriptional activation.^28^^,^^52^ However, the hypoacetylation status of cccDNA-bound H3 and H4 has been correlated with low rates of HBV transcription.^6^ Laura Belloni et al demonstrated that cellular histone acetyltransferases CREB binding protein, p300, and p300/CREB binding protein-associated factor/histone acetyltransferase general control non-depressible 5, and deacetylases histone deacetylase 1 and sirtuin 1 were all recruited to cccDNA *in vivo*,^23^ indicating the complex network of histone acetylation that influences cccDNA transcription. In addition, the E3 ubiquitination ligase Np95/ICBP90-like RING finger protein,^57^ protein kinase S6 kinase 1,^58^ and deacetylase sirtuin 3^52^ reduced the acetylation of histones to inhibit HBV transcription.

In recent years, with the comprehensive application of high-resolution mass spectrometry and biochemical experimental methods, nearly one dozen new histone modification types have been identified, including succinylation, crotonylation, propionylation, malonylation, butyrylation, 2-hydroxybutyrylation, 3-hydroxybutyrylation, and glutarylation. During HBV infection, several novel histone modifications, especially succinylation, have been found to play a crucial role in cccDNA transcription.^59^ General control non-depressible 5, a histone succinyltransferase, promotes cccDNA transcription by modulating succinylation of histone H3 lysine 79.^60^ Furthermore, through our study, we identified two novel succinylation sites, histone H3 lysine 79 and histone H3 lysine 122, on cccDNA, which were regulated by the succinyltransferases lysine acetyltransferase 2A^61^ and deacetylase sirtuin 7,^62^ respectively. Our data showed that the succinylation of histone H3 lysine 79 and histone H3 lysine 122 promoted cccDNA transcription. In addition, Zhao et al were the first to report the de2-hydroxyisobutyrylation of histone H4 lysine 8 on cccDNA minichromosomes mediated by histone deacetylase 3.^32^ These findings have greatly expanded the understanding of histone modifications in cccDNA transcription. Furthermore, we expect the rapid development of technology will lead to increased discoveries of histone modification on cccDNA microchromosomes, which will promote the development of potential epigenetic therapies for HBV infection.

### Role of non-coding RNAs in cccDNA transcriptional modulation

Noncoding RNAs, especially lncRNAs and microRNAs, have been shown to play critical regulatory roles in cccDNA transcription. An increasing number of studies have shown that viral infection may induce the expression of several lncRNAs which subsequently participate in viral transcription and replication.^63^^,^^64^ During HBV infection, elevated lncRNA DLEU2,^65^ proliferating cell nuclear antigen pseudogene 1,^66^ and HULC^67^ levels contribute to cccDNA transcription as well as HBV replication. Our study also revealed that increased levels of the lncRNA HOTAIR promoted HBV transcription by increasing the activity of HBV promoters.^68^ Furthermore, LINC01431 has been shown to play an inhibitory role in cccDNA transcription by increasing the methylation rate and decreasing the acetylation rate of cccDNA.^21^ Altogether, lncRNAs play dual roles in HBV transcription and replication.

MicroRNAs, highly conserved endogenous noncoding RNAs, can regulate both cccDNA quantity and transcription. It has been proven that hsa-miR-138-5p inhibited apolipoprotein B mRNA editing enzyme catalytic subunit 3B-mediated cccDNA decay.^69^ Moreover, miRNA-302c-3p decreased the level of cccDNA by directly interfering with the initiation step of reverse transcription.^70^ In terms of transcription, miRNA-146a indirectly promoted cccDNA transcription by enhancing the expression of the transcription factor structure-specific endonuclease 1.^71^ Furthermore, histone modification has been shown to be involved in microRNA-mediated cccDNA transcriptional regulation. miR-20a increased the methylation of cccDNA-bound histones to suppress cccDNA transcription,^45^ and miRNA-548a might have influenced the deacetylation of cccDNA-bound histones to enhance cccDNA transcription.^72^ Collectively, the effect of microRNAs on cccDNA transcription depends on the downstream axis involved in the mechanism of action.

### Role of HBx in cccDNA transcription

**Figure 2:**
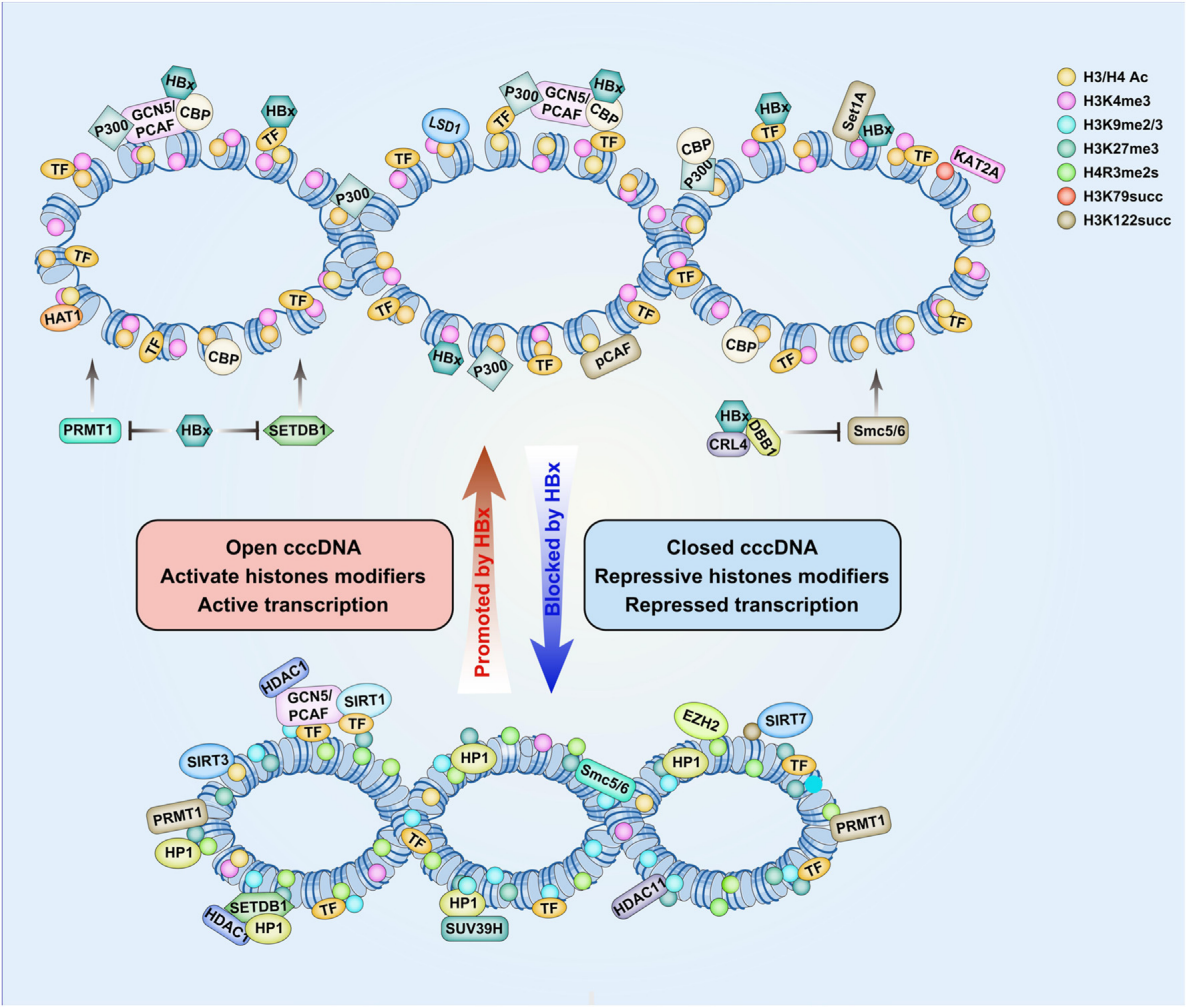
Schematic representation of histone-modifying factor recruitment on cccDNA minichromosomes in relation to viral protein HBx. In the presence of HBx, histone-modifying factors such as p300/CBP, Set1A, and HAT1 are activated and loaded onto cccDNA, resulting in an open configuration of the cccDNA minichromosomes. This open configuration allows viral gene transcription, which leads to a high level of viral replication. Conversely, in the absence of HBx, host restriction factors such as HDAC1, PRMT1, and SIRT3 are loaded onto cccDNA, resulting in a closed configuration of the cccDNA minichromosomes. This closed configuration leads to repression of viral gene transcription, resulting in a low level of viral replication. PRMT1, arginine methyltransferase 1; CBP, CREB binding protein; HDAC1, histone deacetylase 1; SIRT3, sirtuin 3; HAT1, histone acetyltransferase 1.

To counteract the host restriction of cccDNA transcription, HBV has evolved various evasion strategies, of which HBx encoding is the most important. HBx is recruited to cccDNA minichromosomes and the kinetics of HBx recruitment to cccDNA parallel that of HBV transcription.^23^ In the presence of HBx, the number of active histone modifications increases, resulting in the viral minichromosome showing an open configuration, enabling HBV transcription. In contrast, in the absence of HBx, host restriction factors are loaded onto cccDNA, which initiates the acquisition of a suppressive histone-modification state that results in the viral minichromosome being in a closed configuration^6^^,^^23^ (Fig. 2). Increasing evidence has shown that HBx plays an indispensable role in regulating cccDNA transcription by interacting with host factors, such as histone deacetylases, histone acetyltransferases, histone methyltransferases, and demethylases, to produce an active chromatin landscape on cccDNA minichromosomes.^6^^,^^23^^,^^25^^,^^27^^,^^28^^,^^54^ HBx promotes the H3/H4 acetylation of cccDNA by promoting the recruitment of histone acetyltransferases p300/CREB binding protein-associated factor/general control non-depressible 5 and p300/CREB binding protein and inhibiting the binding of histone deacetylase sirtuin 1 and histone deacetylase 1 to cccDNA.^23^ In addition, HBx antagonizes host inhibitory effects on cccDNA transcription induced by histone methyltransferase SET domain bifurcated 1-mediated histone 3 lysine 9 trimethylation, arginine methyltransferase 1-mediated asymmetric dimethylation of histone H4 on arginine 3, and heterochromatin protein 1 recruitment.^21^^,^^27^ HBx can also recruit lysine-specific demethylase 1 and SET domain containing 1A histone lysine methyltransferase to viral promoters, thereby activating viral transcription by inhibiting histone H3 lysine 9 dimethylation and facilitating histone H3 lysine 4 trimethylation, respectively.^73^ In addition, our study confirmed that HBx promotes the transcriptional activity of cccDNA by promoting the recruitment of the histone acetylase sirtuin 1 and histone methylases SET domain containing 1A histone lysine methyltransferase and inhibiting suppressor of variegation 3–9 homolog 1 binding to cccDNA.^52^ Therefore, HBx promotes histone modification that activates cccDNA transcription and inhibits histone modification related to transcriptional inhibition.

In addition to its important role in regulating histone modification via the recruitment of enzymes to cccDNA minichromosomes, HBx may also promote cccDNA transcription by regulating the expression of host factors in a host ubiquitin–proteasome-dependent manner. A well-characterized HBx interactor is cellular DNA damage-binding protein 1.^74^, ^75^, ^76^, ^77^ Several independent studies have confirmed that the DNA damage-binding protein 1-HBx complex promotes the structural maintenance of chromosome 5/6 (SMC5/6) interaction with the cullin 4A-RING E3 ubiquitin ligase, a component of the ubiquitin‒proteasome system, to trigger the ubiquitination and degradation of the SMC5/6 complex, thereby relieving the inhibition of HBV replication.^74^^,^^75^^,^^78^^,^^79^ In addition, HBx has also been shown to induce the ubiquitination and degradation of other antiviral restriction factors, such as SMC6, stromal interaction molecule 1, zinc finger E-box binding homeobox 2, and proteasome activator subunit 4.^80^ Notably, viral HBx protein and host factors, including the SMC5/6 complex, also control transcriptionally activating but not inactivating cccDNA localization in discrete regions of the host genome.^81^^,^^82^ Thus, HBx modulates the epigenetic landscape of cccDNA minichromosomes, and targeting HBx or HBx-associated protein–protein interactions may be an attractive therapeutic strategy for the functional inactivation of cccDNA.

## Antiviral strategies targeting cccDNA transcription

### Targeting transcription factors

HBV cccDNA transcription is controlled by host transcription factors, including ubiquitous transcription factors, liver-enriched transcription factors, and nuclear receptors. Therein, nuclear receptors have been demonstrated to promote cccDNA transcriptional activity by binding to core, pre-S, and X promoters. Notably, researchers generated small molecules that mimic ligands to competitively bind to nuclear receptors and then counteract the activation of HBV transcription via nuclear receptors. For instance, small-molecule retinoic acid receptor agonists, such as tretinoin (all-trans retinoic acid), acitretin, adapalene, and tazarotene, showed profound antiviral effects by repressing the transcription induced by these binding promoters^83^; retinoid X receptor agonist bexarotene inhibited HBV RNA level with an IC_50_ ≤ 5 μM; farnesoid X receptor agonist GW4064 inhibited the expression of HBV RNAs and reduced cccDNA levels in differentiated HepaRG cells^84^; liver X receptor agonists T0901317 and GW3965 have been found to be potent inhibitors of the transcription of viral RNAs in HepaRG cells and primary human hepatocytes but not in human hepatoma HepG2 cells that express sodium taurocholate co-transporting polypeptide (Table 1).^85^ In conclusion, the development of anti-HBV drugs through computer-aided drug design based on the binding affinities between nuclear receptors and ligands may lead to drugs that can be subjected to preclinical research in the future.

**Table 1:** Antiviral strategies targeting cccDNA transcription.

Therapeutic strategy	Drug family	Drug name
Targeting transcription factors	RAR agonists	Tretinoin, Acitretin, Adapalene, Tazarotene
	RXRα agonists	Bexarotene
	FXRα agonist	GW4064
	LXR agonists	T0901317 GW3965
Targeting HBx protein	HBx–DDB1 protein interaction inhibitor	Nitazoxanide
	Pancyclophilin inhibitor	CRV431
	FXR agonist	Vonafexor
Targeting the epigenetic control of cccDNA minichromosomes	histone deacetylase inhibitors (HDACis)	AGK2, IFNα
	histone acetyltransferase inhibitors	C646
	demethylase inhibitors (HDMTs)	GS-5801
Gene editing tool-based epigenetic regulation of cccDNA	zinc finger nucleases (ZFNs)	
	transcription activator-like effector nucleases (TALENs)	
	apolipoprotein B mRNA-editing enzyme catalytic polypeptide-like (APOBEC)	
	CRISPR/Cas system	
Posttranscriptional control	small interfering RNAs (siRNAs)	AB-729, JNJ3989, VIR-2218, HT-101, RG6346
	antisense oligonucleotides (ASOs)	GSK836

### Targeting HBx protein

HBx binds to host restriction factors, such as Smc5/6, sirtuin 3, and arginine methyltransferase 1, and thus counteracts their inhibitory effect on cccDNA. Therefore, developing a therapeutic strategy that disrupts the binding between HBx and restriction factors is a promising direction toward a cure (Table 1). On the basis of this concept, Sekiba et al^86^ found that nitazoxanide, an orally administered thiazolide anti-infective agent, significantly restored Smc5 protein levels and suppressed viral transcription and viral protein production by inhibiting the DNA damage-binding protein 1–HBx protein interaction. Recently, a phase I clinical trial on nitazoxanide was completed, and the results showed that 33% of the subjects (3 of 9) presented with antiviral efficacy and loss of HBsAg.^87^ Unfortunately, the quantitative kinetic analysis of the HBsAg levels in individual subjects did not find any nitazoxanide responders and no increased antiviral efficacy was found when nitazoxanide was combined with nucleos(t)ide analogues. Similarly, neuronal precursor developmentally down-regulated protein 8 is another key factor in the DNA damage-binding protein 1-CUL4-ROC1 E3 ligase pathway, and the neuronal precursor developmentally down-regulated protein 8-activating enzyme inhibitor PEVONEDISTAT has been demonstrated to be a potent inhibitor of HBV because it restored Smc5/6 protein levels.^88^ In addition, a pancyclophilin inhibitor, CRV431, showed significant inhibition of HBV DNA, HBeAg, and HBsAg production in a transgenic mouse model by blocking the formation of the HBx–cyclophilin A complex. A novel, selective nonbile acid, and second-generation FXR agonist, Vonafexor (EYP001),^89^ has been shown to effectively inhibit HBV cccDNA transcription by interfering with the interaction between the nuclear receptor FXR and HBx. Phase IIa clinical trial results showed that combination therapy with Vonafexor and PEG-IFN-α reduced the HBsAg level on average by ≥1 log10 after 16 weeks of treatment in chronic hepatitis B patients. In our previous work, we found that dicumarol significantly inhibited the transcription of HBV cccDNA by targeting HBx and promoting its degradation.^90^ However, clinical research data are lacking.

### Targeting the epigenetic control of cccDNA minichromosomes

Epigenetic therapy that silences cccDNA has shown promise as a therapeutic strategy for a functional cure. Several studies have reported that HBV cccDNA can be differentially methylated, leading to transcriptional repression, reduced viremia, and loss of HBeAg.^91^^,^^92^ In mammals, the DNA methyltransferase family (DNA methyltransferase 1/2/3A/3B) plays an important role in the methylation of structural nucleic acids.^93^ Increased expression of DNA methyltransferases can lead to hypermethylation of viral DNA, a decrease in viral mRNA and protein, and a reduction in HBV replication.^94^, ^95^, ^96^ However, few clinical trials on DNA methylation strategies used in the treatment of HBV infection have been reported, and most of the studies being performed are still in the laboratory research stage.

In terms of histone modification, a number of histone deacetylase inhibitors and demethylase inhibitors have been approved by the FDA as anti-cancer drugs.^97^, ^98^, ^99^ Recently, studies have shown that certain demethylase inhibitors and histone deacetylase inhibitors can suppress cccDNA transcription in tissue cultures without affecting host gene expression (Table 1).^100^^,^^101^ AGK2, a histone deacetylase sirtuin 2 inhibitor, has been shown to repress HBV replication in cell lines and transgenic mice.^102^ GS-5801 is a prodrug of a lysine demethylase-5 inhibitor. Preclinical studies have shown that GS-5801 exerts a comprehensive antiviral effect on HBV-infected primary human hepatocytes.^103^ However, Gilead Sciences terminated a phase I trial on GS-5801 due to the poor gene specificity and toxic side. In addition, C646, a selective small-molecule inhibitor of p300/CREB binding protein histone acetyltransferase, has been demonstrated to induce significant inhibition of HBV transcription without inducing cytotoxicity. Indeed, as a clinically approved antiviral therapy for hepatitis B, interferon has been demonstrated to be related in part to the alteration of cccDNA epigenetic modifications. Zhang et al reported that IFN-α effectively depressed histone H3 lysine 79 succinylation of HBV cccDNA minichromosomes, leading to epigenetic silencing of cccDNA transcription by modulating the activity of the histone acetyltransferase general control non-depressible 5.^60^ Levrero et al found that IFN-α inhibits HBV cccDNA transcription by recruiting human sirtuin 1 and histone deacetylase 1 to reduce histone acetylation. In addition, IFN-α has also been found to inhibit HBV transcription and replication by reducing the binding of signal transducer and activator of transcription 1/2 complexes to cccDNA and inactivating histone acetylation (histone H3 lysine 9 acetylation and histone H3 lysine 27 acetylation) on cccDNA microchromosomes.^104^, ^105^, ^106^ These studies have clearly shown that interferon may inhibit cccDNA transcriptional activity via epigenetic modification, and combining interferon with epidrugs may soon lead to the development of a more potent inhibitor of cccDNA with few side effects.

### Gene editing tool-based epigenetic regulation of cccDNA

Effector domains, such as transcriptional activators, repressors, and chromatin remodeling enzymes, have been found to be capable of being fused with DNA binding domains to facilitate the transcriptional regulation of the epigenome.^107^ Studies have indicated that directly targeting these epigenetic regulatory factors using gene editing tools could potentially overcome the non-specific effects of epigenetic drugs on host gene expression.^108^ Consequently, epigenome engineering has gained popularity as a targeted gene therapy approach to induce locus-specific epigenetic changes for the treatment of genomic diseases. The genetic editing technology has been a promising tool for the inactivation of HBV cccDNA and provided a possibility to actually result in a functional cure of chronic HBV infections. In recent years, the use of designer nucleases for genome editing, such as zinc finger nucleases,^109^ transcription activator-like effector nucleases,^110^ apolipoprotein B mRNA-editing enzyme catalytic polypeptide-like,^111^ and the clustered regularly interspaced short palindromic repeats associated nuclease (CRISPR/Cas) system,^112^ has been employed to target cccDNA (Table 1). However, achieving precise delivery of nucleases to every persistently infected cell is crucial for the success of genetic editing technology. Studies have shown that the most effective result of HBV cleavage using CRISPR/Cas9 is a reduction in cccDNA by approximately 92% *in vitro*.^113^ Additionally, these tools may also produce potential off-target effects, leading to harmful mutations. As a result, the *in vivo* challenge of precise delivery and the off-target effects of the CRISPR/Cas system remain to be addressed.

### Posttranscriptional control

In addition to posttranslational modification, the posttranscriptional regulation of cccDNA is an important link that affects HBV transcription and replication.^114^ At present, two basic approaches targeting HBV mRNA, application of double-stranded small interfering RNAs and antisense oligonucleotides, have been applied to clinical trials.^115^ RNA interference technology is a key method of posttranscriptional regulation of cccDNA. As shown in recent research, there are many small interfering RNAs, including AB-729, JNJ3989, VIR-2218, HT-101, and RG6346, being entered into clinical trials (Table 1). The clinical data indicate that they have markedly decreased the level of HBsAg and HBV DNA and have shown good safety and tolerability profiles.^116^^,^^117^ Antisense oligonucleotides play a therapeutic role in HBV by targeting specific DNA or mRNA sequences and forming hybrid DNA:RNA or RNA:RNA duplexes to degrade targeted RNA and reduce the expression of targeted proteins.^115^ A recently studied antisense oligonucleotide GSK836 has been entered into phase 2b clinical trials and has been shown to reduce all transcripts of cccDNA by targeting all HBV mRNAs as well as common sequences in pregenomic RNA.^118^ However, the use of either antisense oligonucleotides or small interfering RNAs leads to a series of problems, such as toxicity and off-target effects, leading to the termination of clinical trials in the past. These problems need to be solved in the process of developing these classes of drugs.

## Conclusion

Accompany by the mechanism of cccDNA minichromosome formation and regulation gradually being revealed, a large number of host factors and viral proteins have been identified as the modulators of cccDNA activity. On this basis, some small-molecule compounds or strategies targeting cccDNA transcription are being evaluated for their value in clinical applications toward a function-based cure of chronic hepatitis B. Notably, epigenetic modifiers show appealing characteristics and a high potential for decreasing HBV parameters, however, no drugs targeting epigenetic modifications as chronic hepatitis B treatments have been entered into clinical trials. The major reason that these drugs have not advanced past the preclinical research level is the cytotoxic effects and inactivation of certain tumor suppressor genes as well as other unexpected side effects, largely because epigenetic enzymes are involved in many cellular processes.^10^^,^^27^^,^^119^ Therefore, a greater understanding of the specific mechanism of cccDNA minichromosome epigenetic regulation will advance the discovery of drugs specifically targeting the activity of cccDNA.^20^^,^^25^^,^^120^ In addition, the regulatory mechanisms of cccDNA minichromosome assembly and remodeling are not clearly understood. Recently, a study showed that histone regulator A mediated the recruitment of histone variant H3.3 to cccDNA minichromosomes and sustained cccDNA transcriptional activity,^121^ suggesting that understanding the process of cccDNA minichromosome remodeling and identifying specific chromatin remodellers may be beneficial for finding novel antiviral strategies.

In addition to host factors, the crucial role of HBx protein in initiating and maintaining cccDNA transcriptional activity has been confirmed. However, how the original HBx protein products and HBx are recruited to cccDNA minichromosomes needs to be revealed. Compared with host factors, HBx is a safer alternative target for the development of a specific approach to silence the function of cccDNA permanently and block HBx-mediated liver cancer progression.^10^^,^^27^ Therefore, the discovery of designer nucleases, small interfering RNAs, and degradation agents that exclusively reduce HBx protein levels will contribute to blocking cccDNA transcription.^20^^,^^90^^,^^122^ It is also notable that the properties of IFN-α in epigenetic silencing of cccDNA and degradation of HBx protein reveal the potency of combinations of IFN-α, epigenetic modifiers, and anti-HBx agents in the deactivation of cccDNA transcription.^123^^,^^124^

In the near future, combination therapy will constitute the clear trend for functional cure. Recently, these novel therapeutic approaches, involving epigenetic modifiers, targeting HBx compounds, and RNA interference techniques, have been limited to preclinical studies or clinical trials. To apply these approaches in silencing cccDNA transcription in chronic hepatitis B patients to limit off-target effects and hepatotoxicity and to develop specific delivery systems need to be addressed.^125^^,^^126^

## Author contributions

Conceptualization, J.C.; writing—original draft preparation, J.H.R., S.T.C., F.R., and H.Y.G.; writing—review and editing, D.Q.W., X.Y.Y., and M.T. All authors read and agreed to the published version of the manuscript.

## Conflict of interests

Ailong Huang is the member of *Genes & Diseases* Editorial Board. To minimize bias, he was excluded from all editorial decision-making related to the acceptance of this article for publication. The remaining authors declare no conflict of interests.

## Funding

This article was supported by funds from the National Key R&D Program of China (No. 2022YFA1303600), the 10.13039/501100001809National Natural Science Foundation of China (No. 82273423, 82202501), Scientific and Technological Research Program of Chongqing Municipal Education Commission of China (No. KJQN202100429), Natural Science Foundation Project of Chongqing, China (No. CSTB2022NSCQ-MSX0864), and College Young Teachers Fund of the Fok Ying Tung Education Foundation (China) (No. 171100).
